# Serologic evidence for West Nile virus infection in birds in the New York City vicinity during an outbreak in 1999.

**DOI:** 10.3201/eid0704.010403

**Published:** 2001

**Authors:** N Komar, N A Panella, J E Burns, S W Dusza, T M Mascarenhas, T O Talbot

**Affiliations:** Centers for Disease Control and Prevention, PO Box 2087, Fort Collins, CO 80522, USA. nkomar@cdc.gov

## Abstract

As part of an investigation of an encephalitis outbreak in New York City, we sampled 430 birds, representing 18 species in four orders, during September 13-23, 1999, in Queens and surrounding counties. Overall, 33% were positive for West Nile (WN) virus-neutralizing antibodies, and 0.5% were positive for St. Louis encephalitis virus-neutralizing antibodies. By county, Queens had the most seropositive birds for WN virus (50%); species with the greatest seropositivity for WN virus (sample sizes were at least six) were Domestic Goose, Domestic Chicken, House Sparrow, Canada Goose, and Rock Dove. One sampled bird, a captive adult Domestic Goose, showed signs of illness; WN virus infection was confirmed. Our results support the concept that chickens and House Sparrows are good arbovirus sentinels. This study also implicates the House Sparrow as an important vertebrate reservoir host.


### Serologic Evidence for West Nile Virus Infection in Birds in the New York City Vicinity During an Outbreak in 1999

**Nicholas Komar,* Nicholas A. Panella,* Joseph E. Burns,* Stephen W. Dusza,* Tina M. Mascarenhas,* and Thomas O. Talbot†**
*Centers for Disease Control and Prevention, Fort Collins, Colorado, USA; and †New York State Department of Health, Troy, New York, USA

As part of an investigation of an encephalitis outbreak in New York City, we sampled 430 birds, representing 18 species in four orders, during September 13-23, 1999, in Queens and surrounding counties. Overall, 33% were positive for West Nile (WN) virus-neutralizing antibodies, and 0.5% were positive for St. Louis encephalitis virus-neutralizing antibodies. By county, Queens had the most seropositive birds for WN virus (50%); species with the greatest seropositivity for WN virus (sample sizes were at least six) were Domestic Goose, Domestic Chicken, House Sparrow, Canada Goose, and Rock Dove. One sampled bird, a captive adult Domestic Goose, showed signs of illness; WN virus infection was confirmed. Our results support the concept that chickens and House Sparrows are good arbovirus sentinels. This study also implicates the House Sparrow as an important vertebrate reservoir host.

West Nile (WN) virus, a mosquito-borne flavivirus native to Europe, Africa, Asia, and Oceania (1), was first detected in North America in the vicinity of New York City in September 1999 (2,3). The virus was associated with an outbreak that included illness and death in humans (4), horses (5), and birds (6,7). In the Old World, birds serve as the vertebrate reservoir hosts in the transmission cycle of WN virus, while humans and other mammals are incidental hosts (1). The North American counterpart to WN virus is St. Louis encephalitis (SLE) virus. SLE virus is a genetically closely related flavivirus with a similar transmission cycle; it is distributed throughout the Americas (8).

Diagnostic tests for SLE and WN virus infections often cross-react. However, SLE virus had never been detected in New York City, and therefore no arboviral surveillance was in place to recognize a flavivirus epizootic in birds or in mosquitoes. Anecdotal evidence suggested that the WN virus epizootic began in late July 1999, when deaths in crows and other birds were observed in the Queens Borough of New York City and later in other boroughs and surrounding counties. In September 1999, the geographic distribution of WN virus in the New York City area and its natural association with potential mosquito vectors and vertebrate reservoir hosts remained unknown.

To generate basic information on the geographic distribution of WN virus and on its vertebrate host associations in the New York City region, a variety of surveys for flavivirus antibodies were conducted in vertebrate populations there. This report describes one such survey, which targeted resident bird populations in the northeastern quadrant of Queens County, where most of the human WN encephalitis cases were clustered, and in the peripheral counties of Kings (borough of Brooklyn), Richmond (borough of Staten Island), Westchester, and Nassau.

### Methods

### Site Selection

Northeastern Queens was selected as a central sampling location to coincide with the region of greatest density of human WN encephalitis cases (Figure). Three scattered peripheral locations (Valley Stream, Nassau County; New Rochelle, Westchester County; and Staten Island, Richmond County) were selected in which to investigate potential

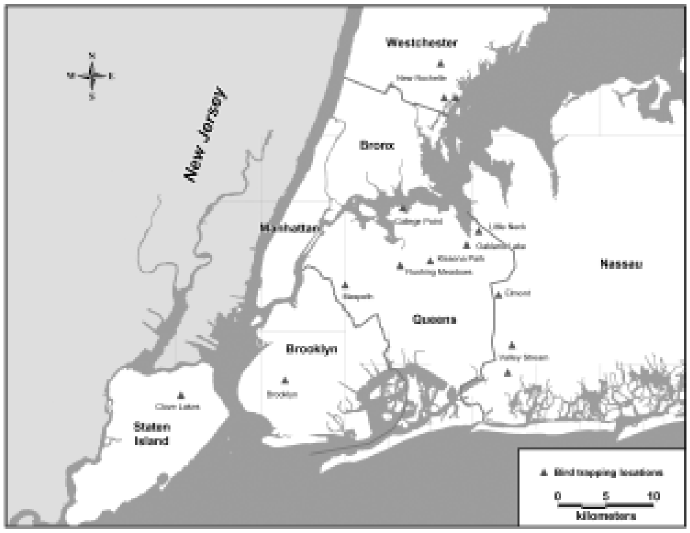

Figure. Bird sampling locations in and around northeastern Queens.

Address for correspondence: Nicholas Komar, Centers for Disease Control and Prevention, PO Box 2087, Fort Collins, Colorado 80522, USA; fax: 970-221-6476; e-mail: nkomar@cdc.gov spread of WN virus transmission away from the apparent epicenter. Samples were collected from Brooklyn, midway between northeastern Queens and Staten Island, because a human case had been reported there. Specific sites within these locations were chosen by convenience, depending on the availability of resident birds for sampling. When possible, captive birds were sampled because residence histories and ages of these birds could be provided by their owners.

### Bird Capture

Wild birds were captured with mist nets (Avinet, Inc.; Dryden, NY), a radio control-operated spring net (Fuhrman Diversified, Inc.; Seabrook, TX), a net gun, or manually when birds were sufficiently tame. Capture of wild birds was authorized by New York State Department of Environmental conservation permit #LCP99-630. Wild birds (but not domestic birds) were marked with uniquely numbered aluminum bands provided by the U.S. Department of Interior Bird Banding Laboratory, as authorized by permit #22866. Use of birds as research subjects for arbovirus seroprevalence studies was registered with the Centers for Disease Control and Prevention (CDC) through Animal Use Protocol #00-26-001-MSA.

### Sample Collection

Whole blood was collected by jugular venipuncture or bracheal venipuncture. The volume of blood collected depended on the size of the bird but did not exceed 0.6 mL. Blood was collected in Microtainer serum collection tubes (Becton Dickinson and Co., Paramus, NJ, USA), held at ambient temperature for at least 15 minutes to permit clotting, and placed into coolers. Each night, serum was separated from blood samples collected earlier in the day by centrifugation with a portable microcentrifuge. Serum was transferred into 2-mL cryovials for shipment to the Division of Vector-Borne Infectious Diseases laboratory, CDC, in Fort Collins, Colorado.

### Virus Strains

The EG101 strain of WN virus, obtained from the CDC reference collection of arboviruses, has a history of 13 unknown passages and 2 passages in suckling mice. The NY99-4132 strain was obtained from the brain of an American Crow (*Corvus brachyrhynchos*) collected in New York during 1999, provided by W.B. Stone. This strain was passaged once in Vero cells before use. The TBH-28 strain of SLE virus was obtained from the CDC reference collection; it has an unknown passage history that includes at least seven passages in suckling mice.

### Plaque Reduction Neutralization Test

Serum samples were screened for flavivirus antibodies in the following manner: Serum samples were heat inactivated for 30 minutes at 56°C to inactivate adventitious microorganisms and nonspecific inhibitors of virus neutralization. Each specimen was diluted 1:5 in a total volume of 75 µL B Buffer (composed of M-199 salts, 1% bovine serum albumin, 350 mg/L sodium bicarbonate, 100 units/mL penicillin, 100 mg/L streptomycin, 1 mg/L Fungizone in 0.05 M Tris, pH 7.6) in sterile 96-well microtiter plates. To these dilutions, we added 75 µL of B buffer that contained approximately 75 Vero PFU of WN virus or SLE virus and 8% normal human serum. The final serum dilution of this mixture was 1:10, and concentration of WN virus was 50 plaque-forming units (PFU)/0.1 mL. The mixture was incubated for 1 hour at 37°C, 5% $CO_2$. Vero cell monolayers grown in six-well culture plates (Costar, Cambridge, MA, USA) were inoculated with 0.1 mL of the serum-virus mixture and incubated for 1 hour at 37°C, 5% $CO_2$. Cells were overlaid with 3 mL per well of 0.5% agarose in M-199 medium supplemented with 350 mg/L sodium bicarbonate, 29.2 mg/L L-glutamine, and antibiotics as in B buffer. After 48 hours of additional incubation, a second 3-mL 0.5% agarose overlay containing 0.004% neutral red dye was added for plaque visualization. Plaques were counted on days 3 and 4 after infection of the Vero cells. Controls included B buffer only (cell viability control), bird serum-free virus mixture with B buffer only (to count PFUs in the challenge dose of virus) and flavivirus (WN or SLE) hyperimmune mouse ascitic fluid (diluted 1:200) mixture with virus (to verify challenge virus identity). Serum samples that neutralized ≥80% of the challenge virus were selected for further titration against both WN virus and SLE virus.

Flavivirus titers of serum samples that tested positive in preliminary screen tests were determined as follows. With the use of 96-well microtiter plates, six serial twofold dilutions of serum in B buffer were prepared beginning with a dilution of 1:5. Virus mixtures were added as described above, resulting in final serum dilutions of 1:10, 1:20, 1:40, 1:80, 1:160, and 1:320. Endpoint titers were assigned as the greatest dilution in which ≥90% neutralization of the challenge virus was achieved. Samples with reciprocal 90% neutralization titers of ≥10 were considered positive. Endpoints for samples with reciprocal titers ≥320 were not determined unless it was necessary to distinguish between WN and SLE viruses as the cause of infection. A sample that showed a fourfold greater titer for one of the viruses was considered positive for neutralizing antibodies to that virus. If a fourfold difference could not be demonstrated, designation as flavivirus-antibody positive was assigned.

### Relative Abundance of Bird Species

To estimate relative abundance of the bird species that we sampled in suburban habitats of northeastern Queens, we relied on subjective estimates of several observers of bird populations in urban and suburban habitats of New York City.

### Statistical Analysis

Pearson chi-square statistics were used to compare seroprevalence percentages (SAS 8.0). If 20% of the expected cell frequencies were <5%, p-values were established by the Fisher exact test. Significance was tested at a level of 0.05.

### Results

We collected serum samples from 430 birds resident in and around northeastern Queens during September 13-23, 1999, and tested them for flavivirus-neutralizing antibodies. Eighteen species, representing four orders, were sampled. Three species comprised 80% of samples (chicken [38%], Rock Dove or Domestic Pigeon [28%], and House Sparrow [16%]). WN virus-neutralizing antibodies were detected in serum from 9 of the 18 species examined, including representatives of all four orders (Table 1). Overall, approximately one third of

Table 1. Flavivirus-neutralizing antibody in birds during September 1999, by species

| Order | Common name | Latin name | Total tested | Percent virus Ab pos. ([95% CI], no.) WN | SLE | FLAV |
|---|---|---|---|---|---|---|
| Anseriformes | Canada Goose | *Branta canadensis* | 16 | 18.8 ([3.9-54.8], 3) | 0 | 6.2 ([0.1-34.8], 1) |
| | Domestic Goose | *Anser* species | 11 | 63.6 ([30.8-89.1], 7) | 0 | 0 |
| | Mallard/Domestic Duck | *Anas platyrhynchos* | 21 | 4.8 ([0.1-26.5], 1) | 0 | 0 |
| | Muscovy Duck | *Cairina moschata* | 1 | 0 | 0 | 0 |
| | Mute Swan | *Cygnus olor* | 1 | 0 | 0 | 0 |
| | Ruddy Shelduck | *Tadorna ferruginea* | 7 | 0 | 0 | 0 |
| | Wood Duck | *Aix sponsa* | 1 | 0 | 0 | 0 |
| Galliformes | Domestic Chicken | *Gallus gallus* | 157 | 56.7 ([48.6-64.6], 89) | 0 | 0 |
| | Common Peafowl | *Pavo cristata* | 10 | 0 | 0 | 0 |
| | Turkey | *Meleagris gallopavo* | 3 | 66.7 ([9.4-99.1], 2) | 0 | 33.3 ([0.8-91.0], 1) |
| Columbiformes | Mourning Dove | *Zenaida macroura* | 3 | 66.7 ([9.4-99.1], 2) | 0 | 0 |
| | Rock Dove | *Columba livia* | 120 | 13.3 ([7.8-20.7], 16) | 0.8 ([0.2-4.6], 1) | 1.7 ([0.2-5.9], 2) |
| Passeriformes | American Robin | *Turdus migratorius* | 1 | 0 | 0 | 0 |
| | Brown-headed Cowbird | *Molothrus ater* | 5 | 40.0 ([5.3-85.3], 2) | 0 | 0 |
| | Common Grackle | *Quiscalus quiscula* | 2 | 0 | 0 | 0 |
| | European Starling | *Sturnus vulgaris* | 2 | 0 | 0 | 0 |
| | House Sparrow | *Passer domesticus* | 67 | 26.9 ([16.8-39.1], 18) | 1.5 ([0.3-8.0], 1) | 3.0 ([0.4-10.4], 2) |
| | Red-winged Blackbird | *Agelaius phoeniceus* | 2 | 0 | 0 | 0 |
| | | | 430 | 32.6 ([28.1-37.2], 140) | 0.5 ([0.05-1.7], 2) | 1.2 ([0.3-2.7], 5) |

Ab: antibody; CI: confidence interval; WN: West Nile; SLE: St. Louis encephalitis; FLAV: flavivirus.

the birds were positive for WN virus-neutralizing antibodies, whereas 0.5% tested positive for SLE virus-neutralizing antibodies. The six species for which >10 birds were sampled each had at least one WN virus-seropositive bird. Of the eight species represented by at least six individuals, the Domestic Goose was the most frequently exposed to flavivirus infection, followed by Domestic Chicken, House Sparrow, Canada Goose, Rock Dove, and Mallard.

Seroprevalence differences for WN virus in birds sampled in different regions were evaluated for each of five New York counties (Table 2). WN virus-infected birds were detected in all five counties, but seroprevalence was greatest in Queens ($\chi^2_{4df} = 92.0$, $p \leq 0.001$). Differences in seroprevalence in the other four counties were not statistically significant ($\chi^2_{3df} = 3.2$, $p \leq 0.364$). A limitation of this analysis is that bird populations sampled may not be representative within each county.

The differences in seroprevalence among species could not be compared across regions where different levels of activity were observed. However, such an analysis was possible within northeastern Queens, where a dozen species were sampled. Again, three species represented approximately 80% of all specimens obtained (Domestic Chicken [56%], Rock Dove [19%], and House Sparrow [8%]). WN virus-neutralizing antibodies were detected in serum from 9 of the 12 species examined; half of these had seroprevalences of ≥50% (Table 3). Sample sizes were adequate to allow comparison of four species. From this analysis, Domestic Chickens and House Sparrows were the most frequently infected with WN virus; Mallards were least frequently infected; and Rock Doves were intermediate.

We evaluated cross-reactivity between WN and SLE viruses by the plaque reduction neutralization test (PRNT). The two specimens that were positive for SLE virus-neutralizing antibodies were negative for WN virus antibodies in the initial screen assay. However, of 140 WN virus antibody-positive specimens tested for SLE antibodies, 9 (6.4%) had 90% neutralization titers of ≥20 for SLE. Typically, WN virus antibody titers were more than eightfold greater than SLE titers, but this finding depended on the strain of WN virus used in the PRNT.

One of the captive birds sampled, an adult male Swan Goose (*Anser chinensis*, a type of Domestic Goose), was recovering from an illness characterized by ataxia at the time

Table 2. Flavivirus-neutralizing antibody detected in birds during September 1999, by county

| County (NY) | Total tested | Percent virus Ab pos. ([95% CI], no.) WN | SLE | FLAV |
|---|---|---|---|---|
| Queens | 253 | 50.1 ([44.3-56.9], 128) | 0 | 1.2 ([0.2-3.5], 3) |
| Richmond | 43 | 2.3 ([0.1-12.3], 1) | 0 | 0 |
| Kings | 20 | 5.0 ([0.1-37.5], 1) | 0 | 0 |
| Nassau | 61 | 6.6 ([1.8-15.9], 4) | 1.6 ([0.04-8.8], 1) | 1.6 ([0.04-8.8], 1) |
| Westchester | 53 | 11.3 ([4.3-23.0], 6) | 1.9 ([0.04-10.0], 1) | 3.8 ([0.5-13.0], 2) |

Ab: antibody; CI: confidence interval; WN: West Nile; SLE: St. Louis encephalitis; FLAV: flavivirus.

Table 3. Flavivirus-neutralizing antibody in birds in Queens during September 1999, by species

| Common Name | Total tested | Percent virus Ab pos. ([95% CI], no.) WN | FLAV |
|---|---|---|---|
| Canada Goose | 7 | 28.6 ([3.6-71.0], 2) | 14.2 ([0.3-57.4], 1) |
| Domestic Goose | 7 | 85.7 ([42.1-99.6], 6) | 0 |
| Mallard/Domestic Duck | 16 | 6.3 ([0.2-34.8], 1) | 0 |
| Domestic Chicken | 141 | 63.1 ([54.6-71.1], 89) | 0 |
| Turkey | 3 | 66.7 ([9.4-99.2], 2) | 33.3 ([0.8-90.6], 1) |
| Mourning Dove | 1 | 100.0 ([2.5-100.0], 1) | 0 |
| Rock Dove | 49 | 26.5 ([14.9-41.1], 13) | 2.0 ([0.05-11.4], 1) |
| American Robin | 1 | 0 | 0 |
| Brown-headed Cowbird | 4 | 50.0 ([6.8-93.2], 2) | 0 |
| House Sparrow | 20 | 60.0 ([36.1-80.9], 12) | 0 |
| European Starling | 2 | 0 | 0 |
| Red-winged Blackbird | 2 | 0 | 0 |

Ab: antibody; CI: confidence interval; WN: West Nile; FLAV: flavivirus.

of sampling. Its owners were able to provide convalescent-phase serum samples from this bird. WN virus-neutralizing antibody titers for these samples increased from a reciprocal titer of 10 in the acute-phase specimen to 40 in the convalescent-phase specimen, confirming WN virus infection. This is the first confirmed case of WN virus disease in a Domestic Goose in North America.

Relative abundance of bird species, in concert with seroprevalence, is needed to identify candidate avian reservoir hosts for WN virus. We estimated the relative abundances of the six species for which at least seven birds were surveyed in Queens (Table 4). From this analysis, we estimated that House Sparrows contributed 82% to 97% of all WN virus infections in these six species. Rock Doves contributed 3% to 16%, and the other four species contributed negligibly to the total number of infections.

Table 4. Estimated relative abundance of six bird species with West Nile virus seroprevalence and estimated relative number of infections, suburban northeastern Queens

| Bird species | Relative abundance | WN virus Ab prevalence [95% CI] | Relative no. of infections (%)[a] | Percentage range[b] |
|---|---|---|---|---|
| House Sparrow | 6,000 | 0.60 [0.36-0.81] | 4186 (92) | 82-97 |
| Rock Dove | 1,000 | 0.27 [0.15-0.41] | 314 (7) | 3-16 |
| Mallard | 60 | 0.06 [0.002-0.35] | 4 (<1) | <1-<1 |
| Canada Goose | 60 | 0.29 [0.04-0.71] | 20 (<1) | <1-2 |
| Domestic Chicken | 3 | 0.63 [0.55-0.71] | 2 (<1) | <1-<1 |
| Domestic Goose | 1 | 0.86 [0.42-1.00] | 1 (<1) | <1-<1 |

Ab: antibody; CI: confidence interval.
[a]Adjusted relative to Domestic Goose.
[b]This range is determined as follows for each species. For lower bound, the lowest bound of the seroprevalence CI is used to estimate the total relative number of infections; the upper bound of this CI is used for all other species. The converse is assumed for the calculation of the upper bound of the percentage.

### Conclusion

In our study, we investigated seroprevalence for WN virus in resident birds in New York City during September 1999. Seropositive birds were widely spread throughout the New York City region, and local transmission was documented in all five counties surveyed. However, transmission was significantly greater in certain neighborhoods (e.g., northeastern Queens). Comparing the seroprevalences in bird species at one such focus (northeastern Queens), we identified several species of birds that were frequently exposed and that thus could be useful sentinels or important reservoir hosts in the WN virus transmission cycle. Geese, chickens, House Sparrows, and Rock Doves in Queens all had high-level seroprevalences, consistent with the exposure of these species to WN virus in the Romanian outbreak of 1996 (9). These species should be considered for use as captive or free-ranging sentinels for WN virus activity.

Vertebrate seroprevalence data may provide clues to the identity of important reservoir hosts. An important reservoir for WN virus must be abundant relative to other bird species, frequently exposed to infection, and biologically capable of infecting hematophagous arthropods (10). Although we did not directly evaluate abundance or competence, we estimated relative abundance (Table 4). Other studies have evaluated competence of various birds experimentally infected with the New York strain of WN virus. Chickens were unable to develop sufficient viremia to infect large proportions of *Culex* mosquitoes that feed on them (11-13). Although 3-week-old Domestic Geese (*A. anser*) develop infectious-level viremia (14), adult Canada Geese were incompetent (CDC, unpub. data). Rock Doves were similarly incompetent, but House Sparrows maintained infectious-level viremia for several days (CDC, unpub. data). Thus, of the species we evaluated for seroprevalence, the House Sparrow was an important reservoir host because of its abundance, high seroprevalence, and biological competence.

Although some abundant species such as House Sparrow and Rock Dove were well represented in our survey, others were not, such as several icterid species (blackbirds), European Starling, American Robin, and American Crow. Crows were noticeably absent from our study sites and may have been locally extirpated by WN virus. Further studies are required to generate estimates of seroprevalence in these abundant resident bird species.

Seroprevalence data in birds may be difficult to interpret. To rule out alternative flavivirus infection, the birds sampled in our study were tested for antibodies to both WN and SLE viruses. As a result, we detected evidence of SLE (but not WN) virus infection in two resident birds: an adult House Sparrow from New Rochelle (Westchester County) and a 1-year-old captive pigeon in Valley Stream (Nassau County). We also collected age data on the birds sampled and found that numerous seropositive birds were aged as "hatching year" birds, thus confirming that transmission occurred in the current year. We did not have an adequate sample of birds of a single species of different known ages to evaluate whether the seroprevalence patterns in age categories fit an epizootic rather than an enzootic pattern.

Our seroprevalence data should be interpreted with caution. The main conclusions are 1) birds were heavily exposed to WN virus in certain locations in New York City (e.g., northeastern Queens); 2) at least some, if not all, WN virus activity in northeastern Queens occurred in 1999; 3) certain species such as geese, chickens, House Sparrows, and Rock Doves were frequently infected and are likely to serve as effective WN virus sentinels in urban transmission foci; and 4) House Sparrows in particular served as hosts for most of the avian WN virus infections in the bird populations we sampled in northeastern Queens and appear to be an important reservoir host there.

### Acknowledgments

We thank the New York City Urban Parks Department, especially A. Brash and T. Gluck; private property owners who granted permission for work on their premises; R. Cook and M. Rush (Wildlife Conservation Society), J. Charros and C. Vargas (veterinary assistance); V. Gattulo and D. Wanamaker (Staten Island Zoological Society); and R. Smith, V. Mattucci, A. Block, and H. Fischer.

Dr. Komar is the vertebrate ecologist for the Centers for Disease Control and Prevention's Arbovirus Diseases Branch, Division of Vector-Borne Infectious Diseases, Fort Collins, Colorado. His major research interest is in understanding the role of vertebrate hosts in arbovirus transmission cycles.

### References

1. Komar N. West Nile viral encephalitis. Rev Sci Tech 2000;19:166-76.
2. Lanciotti RS, Roehrig JT, Deubel V, Smith J, Parker M, Steele K, et al. Origin of the West Nile virus responsible for an outbreak of encephalitis in the northeastern United States. Science 1999; 286:2333-7.
3. Anderson JF, Andreadis TG, Vossbrinck CR, Tirrell S, Wakem EM, French RA, et al. Isolation of West Nile virus from mosquitoes, crows, and a Cooper's Hawk in Connecticut. Science 1999;286:2331-3.
4. Nash D, Mostashari F, Fine A, Miller J, O'Leary D, et al. The outbreak of West Nile virus infection in the New York City Area in 1999. N Engl J Med 2001;344:1807-14.
5. Office International des Epizooties. West Nile fever in the United States of America in horses. Disease Information 1999;12:150-1.
6. Steele KE, Linn MJ, Schoepp RJ, Komar N, Geisbert TW, Manduca RM, et al. Pathology of fatal West Nile virus infections in native and exotic birds during the 1999 outbreak in New York City, New York. Vet Pathol 2000;37:208-24.
7. Eidson M, Komar N, Sorhage F, Nelson R, Talbot T, Mostashari F, et al. Crow deaths as a sentinel surveillance system for West Nile virus in North America, 1999. Emerg Infect Dis 2001;7:615-620.
8. Monath TP, editor. Saint Louis encephalitis. Washington: American Public Health Association; 1980.
9. Savage HM, Ceianu C, Nicolescu G, Karabatsos N, Lanciotti R, Vladimirescu A, et al. Entomologic and avian investigations of an epidemic of West Nile fever in Romania in 1996, with serologic and molecular characterization of a virus isolate from mosquitoes. Am J Trop Med Hyg 1999;61:600-11.
10. Scott TW. Vertebrate host ecology. In: Monath TP, editor. The arboviruses: Epidemiology and ecology. Vol I. Boca Raton (FL): CRC Press; 1988. p. 257-80.
11. Turell MJ, O'Guinn M, Oliver J. Potential for New York mosquitoes to transmit West Nile virus. Am J Trop Med Hyg 2000;62:413-4.
12. Senne DA, Pedersen JC, Hutto DL, Taylor WD, Schmitt BJ, Panigrahy B. Pathogenicity of West Nile virus in chickens. Avian Dis 2000;44:642-9.
13. Langevin SA, Bunning M, Davis B, Komar N. Experimental infection of chickens as candidate sentinels for West Nile virus. Emerg Infect Dis 2001;7:726-9.
14. Swayne DE, Beck JR, Smith C, Shieh W, Zaki S. Fatal encephalitis and myocarditis in young domestic geese (*Anser anser domesticus*) caused by West Nile virus. Emerg Infect Dis 2001;7:751-3.
